# Is it time to rethink how we page physicians? Understanding paging patterns in a tertiary care hospital

**DOI:** 10.1186/s12913-019-4844-0

**Published:** 2019-12-23

**Authors:** Luke Witherspoon, Emily Nham, Hamidreza Abdi, Ali Dergham, Thomas Skinner, J. Stuart Oake, James Watterson, Luke T. Lavallée

**Affiliations:** 1 Division of Urology, Department of Surgery, The Ottawa Hospital and University of Ottawa, Ottawa, Canada; 2 Faculty of Medicine, University of Ottawa, Ottawa, Canada; 3 Faculty of Health Sciences, School of Medicine, Queen’s University, Kingston, Canada

**Keywords:** Medical education, Medical residency, Physician burnout, Physician paging

## Abstract

**Background:**

Frequent pages can disrupt workflow, interrupt patient care, and may contribute to physician burnout. We hypothesized that paging volumes followed consistent temporal trends, regardless of the medical or surgical service, reflecting systems based issues present in our hospitals.

**Methods:**

A retrospective review of the hospital paging systems for 4 services at The Ottawa Hospital was performed. Resident paging data from April 1 to July 31, 2018 were collected for services with a single primary pager number including orthopaedic surgery, general surgery, neurology, and neurosurgery. Trends in paging volume during the 4-month period were examined. Variables examined included the location of origin of the page (emergency room vs. inpatient unit), and day/time of the page.

**Results:**

During the study period, 25,797 pages were received by the 4 services, averaging 211 (± Standard Deviation (SD) 12) pages per day. 19,371 (75%) pages were from in-patient hospital units, while 6426 (24%) were pages from the emergency room. The median interval between pages across all specialties was 22:30 min. Emergency room pages peaked between 16:30 and 20:00, while in-patient units peaked between 17:30 and 18:30.

**Conclusions:**

Each service experienced frequent paging with similar patterns of marked increases at specific times. This study identifies areas for future study about what the factors are that contribute to the paging patterns observed.

## Background

Numeric hospital paging systems were introduced in the 1970s to facilitate rapid and reliable communication between health care workers. Although they are thought to improve communication and health care delivery, no data exists to support this. Of the limited studies on paging volumes, most have shown that a large proportion of pages are non-urgent and are not associated with patient volumes or care needs [1, 2]. Unnecessary pages may cause harm, as frequent paging has been associated with increased medical and surgical errors [3, 4].

Residency training is comprised of heavy workloads, mental and physical fatigue, and psychological stress. The Royal College of Physicians and Surgeons of Canada have recently made recommendations to decrease resident duty hours in order to promote resident well-being, enhance patient health and avoid errors [5]. Some reported stressors in residency training include long work hours, poor organization, interruption of normal sleep cycles, and hospital pagers [6–9]. Physicians who spend more time on clerical duties report more symptoms of burnout, and burnout has been associated with worse patient outcomes, decreased patient satisfaction, and increased malpractice claims [10, 11].

Small studies have shown that a significant portion (30–50%) of pages to physicians are non-urgent, but a study evaluating the temporal trends of these pages has yet to be done on a large scale in Canada [2, 12, 13]. Through determining patterns and trends, it may be possible to better identify and manage times of high work demands, whether it be through the management of hospital resources and personnel, through potential workload reductions, or both. The aim of this study is to provide insight to paging patterns in a Canadian teaching hospital. These data may be used to guide quality improvement programs or policies aimed at improving communication between health care professionals, to reduce physician stress, and minimize disruptions in patient care and learning opportunities for trainees.

## Methods

### Study design

A retrospective review was conducted to assess the temporal trends in resident paging volume across three surgical (orthopaedic surgery, general surgery, and neurosurgery) and one medical (neurology) service at The Ottawa Hospital (TOH). These services were chosen as they were the only services with a single dedicated service pager. A dedicated service pager is a pager device handed off between the on-call residents, and is the primary first contact pager for a particular medical or surgical service. Within our hospital system only a few services employ dedicated resident pagers. Pages requested for a specific physician, or pages originating outside the hospital were not captured in this study.

All numerical pages sent through the TOH contact centre were captured between April 1 and July 31, 2018. Electronic records of pages are kept for 3 months at our institution, allowing the prior 3 months of data to be accessed. Within our internal software, pages are organized by time, originating extension number, and receiving pager. This data is organized and stored using Microsoft excel. Data collection occurred over the course of July 2018, allowing the month of July to be included in this analysis. Pages to orthopaedic and general surgery were captured at two TOH campuses (Civic and General campus), while the neurosurgery and neurology services are primarily located at a single campus (Civic).

### Statistical analysis

For each page received by the dedicated pagers the date, time, and incoming extension number were determined. Based on the incoming extension number, pages were divided into two groups: pages sent from the emergency room (ER) or pages sent from other extension numbers (in-patient locations).

Total number of pages for all services were calculated stratified by ER or in-patient locations. For each service the mean and standard deviation of total pages per month and day of week were calculated. Mean and median intervals between each page were calculated. To assess the daily time trends in paging and peak paging times, each page’s logged time was rounded to the nearest half-hour. The number of pages falling into each of these half hour segments were then counted. Rates were then calculated along with Poisson 95%-confidence intervals. Peak times were identified by the study team as periods where the rates and associated confidence intervals were statistically greater than non-peak times.

Similarly, paging rates for days of the week as well as months, were calculated along with their associated Poisson 95%-confidence intervals. Comparisons of these confidence intervals were used to establish statistical significance.

All calculations were performed on Microsoft Excel 2016.

### Ethics approval

This study was completed with approval from the institutional ethics board of The Ottawa Hospital.

## Results

### Paging volumes

During the 4-month period, a total of 25,797 pages were made to residents with dedicated pagers in orthopaedic surgery, general surgery, neurology, and neurosurgery, averaging 211 (± standard deviation (SD) 12) calls per day (Table 1). Of the 25,797 pages made, 19,371 (75%) were sent from in-patient locations, while 6426 (25%) were from ER extensions. On average, neurosurgery had the highest number of pages per month at 1499.75 (± 137), while neurology had the least at 531 (± 24). Although Saturday had the greatest rate of pages across all specialties, there was no statistically significant difference when comparing to all other days of the week. Wednesdays were found to have the fewest total number of pages, although it did not appear to have statistically significant differences when comparing to all other weekdays, except when comparing directly to Thursday and Saturday where a significant difference was noted (*p* < 0.05). Comparing the total number of pages per month, April had the fewest rate of pages when compared to the three other months studied (*p* < 0.05). Orthopaedic surgery and neurosurgery services at the Civic campus saw a significant increase in paging volumes in July as compared to June (*p* < 0.05 for both).

**Table 1:** Number of pages received by 4 services at The Ottawa Hospital General (G) and Civic (C) campus

	Total	In-patient locations (% total)	ER (% total)	Mean per month (± SD)	Mean per day (± SD)
General Surgery (C)	5561	4147 (75%)	1414 (25%)	1390 (± 146)	45 (± 4)
General Surgery (G)	5269	3714 (70%)	1555 (30%)	1317 (± 166)	43 (± 2)
Neurology (C)	2124	1864 (88%)	260 (12%)	531 (± 23)	17 (± 5)
Neurosurgery (C)	5999	5033 (84%)	966 (16%)	1500 (± 137)	49 (± 3)
Orthopaedic Surgery (C)	3631	2463 (68%)	1168 (32%)	908 (± 129)	30 (± 3)
Orthopaedic Surgery (G)	3213	2150 (67%)	1063 (33%)	803 (± 108)	26 (± 2)
All Services	25,797	19,371 (75%)	6426 (25%)	6449 (± 1955)	211 (±12)

### Paging frequency

The mean (± SD) interval of time between pages across all specialties was 0:46:19 (± 1:17:51) minutes (Table 2). Between the hours of 16:00 and 20:00, the mean interval decreased to 0:37:55 (± 2:20:12) minutes. Mean duration between pages during potential sleeping hours from 00:00 to 06:00 increased to 1:19:30 h (± 0:50:27).

**Table 2:** Duration of time between pages received by 4 services at The Ottawa Hospital General (G) and Civic (C) campus

	Mean duration			Median Duration
	All pages (± SD)	Pages between 4 pm - 8 pm (± SD)	Pages between 12 am - 6 am (± SD)	All pages
General Surgery (C)	0:31:36 (± 1:30:3)	0:21:55 (± 0:23:51)	0:50:27 (± 0:50:45)	0:18:00
General Surgery (G)	0:33:20 (± 1:04:36)	0:21:43 (± 0:23:41)	1:02:37 (± 0:54:31)	0:18:00
Neurology (C)	1:21:20 (± 3:34:25)	1:42:22 (± 3:31:54)	1:39:35 (± 0:43:19)	0:35:00
Neurosurgery (C)	0:29:04 (± 1:22:21)	17:48 (± 0:39:11)	0:55:22 (± 1:08:10)	0:15:00
Orthopaedic Surgery (C)	0:48:18 (± 1:42:57)	29:48 (± 0:35:08)	1:34:35 (± 0:44:48)	0:24:00
Orthopaedic Surgery (G)	0:54:13 (± 1:51:18)	0:33:56 (± 1:03:10)	1:54:22 (± 0:41:08)	0:25:00
All Specialties	0:46:19 (± 1:17:51)	0:37:55 (± 2:20:12)	1:19:30 (± 0:50:27)	0:22:30

**Fig. 1:**
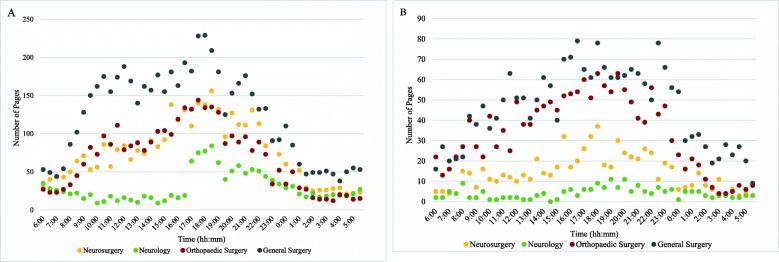
**a** In-patient pages received from April 1st to July 31st according to the time of day. Neurosurgery and neurology values reflect paging volumes at single campus (Civic campus). General surgery and orthopaedic surgery values reflect total paging volume across both campuses (General and Civic campus). **b** ER pages received from April 1st to July 31st according to the time of day. Neurosurgery and neurology values reflect paging volumes at single campus (Civic campus). General surgery and orthopaedic surgery values reflect total paging volume across both campuses (General and Civic campus)

**Fig. 2:**
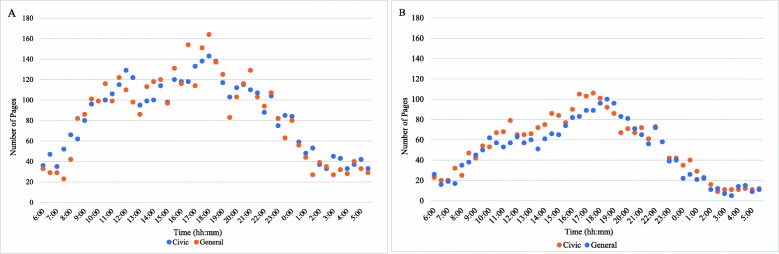
**a** Total pages received by general surgery at two campuses from April 1st to July 31st. **b** Total pages received by orthopaedic surgery at two campuses from April 1st to July 31st

Trends in paging patterns from ER and in-patient locations were similar for all services, with marked increases in average paging volumes from 16:30 to 20:00 (Table 3). Peak paging time ranged from 17:30 to 18:30 for in-patient location pages and 16:30 to 20:00 for ER pages. Paging volumes to all specialties between 16:00 to 20:00 were different than all other times of the day (*p* < 0.01). Paging patterns from in-patient locations are often bimodal with a second peak occurring between 10:00 to 12:00 (Fig. 1a). ER pages are more variable with all services having peaks between 18:00 to 20:00, and smaller peaks throughout the morning and early afternoon (Fig. 1b). Both general and orthopaedic surgery had additional peaks in paging volumes at midnight. Analysis of paging frequency between services offered at both campuses revealed similar trends in peak times for pages (Fig. 2), however not all of these peak times were statistically different from the mean.

**Table 3:** Peak paging times of 4 services at The Ottawa Hospital General (G) and Civic (C) campus

	In-patient locations	ER
General Surgery (C)	17:30:00	16:30:00
General Surgery (G)	18:00:00	18:00:00
Neurology (C)	18:30:00	19:00:00
Neurosurgery (C)	18:30:00	18:00:00
Orthopaedic Surgery (C)	17:30:00	16:30:00
Orthopaedic Surgery (G)	18:30:00	20:00:00
All Specialties	18:05:00	18:00:00

## Discussion

This study shows that there are similar patterns of pages across one medical and three surgical specialities, and across different hospital campuses with different hospital wards and allied health staff. This suggests that these patterns are attributable to system wide processes. Our data shows that residents receive surges in paging volumes at certain times during the day, that are reproducible across the days of the week, and over several months.

An analysis of the temporal trends of pages may provide insight to their likely causes. It was determined that the highest frequency in paging occurred between 16:00 and 20:00 for ER pages and 17:30 and 18:30 for in-patient location pages, although ER paging patterns were more variable than pages received from in-patient locations (Figs. 1b). A report about ER visits in Canadian hospitals from 2016 to 2017 revealed that patient volumes begin to increase at 08:00, peak at 12:00, and gradually decrease towards midnight [14]. This discrepancy between timing of known surges in patient visits and our observed surges in paging volume suggests there may be another cause other than patient-related events. Interestingly, previous studies have shown that there is actually an inverse relationship between patient volumes on the wards and paging frequency [1]. It is unclear why a trend towards less pages on Wednesdays was observed. Perhaps Wednesday’s represent a day when patient issues occurring over the prior weekend have been resolved, thus necessitating less communication between health professionals. Further evaluation of this finding is needed to see if this is consistent across centres and over time. If this finding is consistent this may help hospital administrators and health care teams develop strategies to allocate resources during a weekly cycle or improve access for communication on high volume days.

A study by Patel et al. determined that general surgery interns received a marked increase in pages corresponding to changes in nursing shifts [1]. In our study, in-patient location pages consistently peaked between 17:30 and 18:30, corresponding to TOH nursing shift changes before 19:00. With the assumption that the majority of pages coming from ER extension are requests for consultation or between-unit handoffs, we would expect to see unpredictable paging patterns. Instead, what we observe are consistent paging patterns over the duration of 4 months that potentially correlate with within-unit handoffs and shift changes. Managing in-patient unit resources during hand-over of care or improving communication between health care professionals during shift changes may help to better manage pages of this nature.

In our study, residents were paged on average every 0:37:55 (± 2:20:12) minutes during peak hours between 16:00 and 20:00 and every 1:19:30 (± 0:50:27) hours during potential sleeping time (00:00 to 06:00). Although the activity of residents was not recorded in this study, previous studies determined that frequent paging interrupts patient care activities such as taking medical histories, completing patient charts, performing physical examinations, and completing surgical procedures [2, 12, 13, 15]. Interruptions have been associated with an increased risk of errors in drug dispensing, and surgical procedures [3, 4]. Other times residents were often paged included during scheduled rounds, educational sessions, and basic human activities such as eating, sleeping, and bathroom use [2, 12].

Physicians spend more time on clerical tasks than developing surgical skills or providing direct patient care [16–18]. It has been reported that physicians who perform more clerical duties are less likely to be satisfied with their career, and are more likely to experience symptoms of burnout [10]. Physician burnout is a well-documented problem within the medical system with one in every three physicians experiencing symptoms of depersonalization, emotional exhaustion, and depression [19–21]. A systematic review and meta-analysis found that physician burnout was associated with increased adverse medical events resulting from delivery of care, poor physician-patient communication, low professionalism exhibited by physicians, decreased patient-reported satisfaction, and increased malpractice claims [11].

With the numerical paging system at the study hospital, residents do not have the ability to determine the urgency of calls and must respond immediately to all pages received. A study by Katz and Schroeder determined that 26% of pages received by interns on medical services were not clinically indicated, while another 16% could have been postponed for more than an hour [2]. Additionally, a study of pages received by on-call junior neurosurgery residents found that residents spent 15 to 174 s attending to individual pages [12]. Extrapolating from this study, this would suggest between 3 to 39 days of resident time were spent returning pages from in-patient locations over the course of our study period. Responding to pages is an integral part of resident duties, however when paging volumes are high, it may take time away from other important clinical duties or basic human activities.

Teaching hospitals have an inherent cyclical turnover of physicians, typically in July, when the most experienced resident physicians complete their training, and new resident physicians begin their training. Given that new residents begin training on July 1, we sought to analyze paging data before and after July. If paging volume was to increase in July, this could suggest that the experience of a physician, and the care they provide, has an impact on paging frequency. Interestingly, paging volumes only increased from June to July for orthopaedic surgery and neurosurgery at a single campus. This would seem to suggest that physician inexperience is not a main driver of paging volumes.

It has previously been reported that the majority of incoming pages are non-urgent and result in no changes to patient management [2]. Before interventions to reduce non-urgent or unnecessary pages are employed, perceptions of medical urgencies must be similar across all hospital personnel in order to maximize patient safety. Understandably, it is a difficult task to triage the urgency of clinical situations, after all, pagers were invented to improve communication and collaboration amongst health care professionals. Therefore, managing the timing of pages may be an alternative solution.

Wieland et al. analyzed the efficacy of a triaging system where a chief resident answered pages on behalf of internal medicine residents during 30-min educational sessions [22]. Out of 884 pages made, 743 were identified as non-urgent by the chief resident and could be responded to after the end of sessions. This resulted in an average of 4.22 less pager interruptions per session, providing residents with valuable educational time. In this present study, only data from numerical pagers were included and the urgency of each page could not be determined. Alphanumeric pagers have been one solution to triaging calls and addressing the inefficiencies of numerical paging systems; however, studies suggest that there are still communication break-downs requiring time and attention of residents [23].

### Strengths and limitations

This retrospective study assessed paging patterns for four different services, 3 surgical and 1 medical at a large tertiary care hospital with two campuses over the course of 4 months. This data has previously been presented at a national urological meeting in Canada [24]. Although we were not able to compare pages received from the neurology and neurosurgery service at both hospitals, the data from orthopaedic and general surgery suggest that both hospital campuses experience similar paging trends.

We only included data from specialties with service dedicated numerical pagers. Many medical and surgical specialities at our institution use physician specific pagers, rather than handing over a dedicated service pager. This likely means there are pages being received by personal pagers by physicians on these services which would not have been captured in our study and this could lead to an underestimate of the true paging volume. This means that residents are likely interrupted from their work at a frequency surpassing what this study presents. We were not able to determine the importance/urgency of the pages received. Furthermore, our electronic collection system did not allow for determination of any missed or repeated pages. This could lead to an overestimation of the number of number of pages received by a service. We were only able to analyze paging volume over a 4-month period. It is possible that the trends observed in this study, are not representative of the entire year.

## Conclusion

This retrospective study reveals paging patterns from four medical and surgical specialties across two campuses of The Ottawa Hospital. All services received a high number of pages during the four-month study period. Each service had a similar paging pattern with marked increases in pages at specific times during the day. These data suggest the need to effectively manage paging volumes in order to maximize the efficiency of communications, workflow, resident well-being, resident learning, and patient care. Future research should examine the utility and efficacy of various interventions, including page-triaging systems, two-way alphanumerical pagers, and mobile devices.

## Data Availability

The datasets used and/or analysed during the current study are available from the corresponding author on reasonable request.

## Abbreviations

CUA: Canadian Urology Association Conference
ER: Emergency Room
SD: Standard Deviation
TOH: The Ottawa Hospital
